# Multigene Phylogeny of Choanozoa and the Origin of Animals

**DOI:** 10.1371/journal.pone.0002098

**Published:** 2008-05-07

**Authors:** Kamran Shalchian-Tabrizi, Marianne A. Minge, Mari Espelund, Russell Orr, Torgeir Ruden, Kjetill S. Jakobsen, Thomas Cavalier-Smith

**Affiliations:** 1 Microbial Evolution Research Group, Department of Biology, University of Oslo, Oslo, Norway; 2 Centre for Ecological and Evolutionary Synthesis, University of Oslo, Oslo, Norway; 3 Scientific Computer Group, Center for Information Technology Services, University of Oslo, Oslo, Norway; 4 Department of Zoology, University of Oxford, Oxford, United Kingdom; Texas A&M University, United States of America

## Abstract

Animals are evolutionarily related to fungi and to the predominantly unicellular protozoan phylum Choanozoa, together known as opisthokonts. To establish the sequence of events when animals evolved from unicellular ancestors, and understand those key evolutionary transitions, we need to establish which choanozoans are most closely related to animals and also the evolutionary position of each choanozoan group within the opisthokont phylogenetic tree. Here we focus on *Ministeria vibrans*, a minute bacteria-eating cell with slender radiating tentacles. Single-gene trees suggested that it is either the closest unicellular relative of animals or else sister to choanoflagellates, traditionally considered likely animal ancestors. Sequencing thousands of *Ministeria* protein genes now reveals about 14 with domains of key significance for animal cell biology, including several previously unknown from deeply diverging Choanozoa, e.g. domains involved in hedgehog, Notch and tyrosine kinase signaling or cell adhesion (cadherin). Phylogenetic trees using 78 proteins show that *Ministeria* is not sister to animals or choanoflagellates (themselves sisters to animals), but to *Capsaspora*, another protozoan with thread-like (filose) tentacles. The *Ministeria*/*Capsaspora* clade (new class Filasterea) is sister to animals and choanoflagellates, these three groups forming a novel clade (filozoa) whose ancestor presumably evolved filose tentacles well before they aggregated as a periciliary collar in the choanoflagellate/sponge common ancestor. Our trees show ichthyosporean choanozoans as sisters to filozoa; a fusion between ubiquitin and ribosomal small subunit S30 protein genes unifies all holozoa (filozoa plus Ichthyosporea), being absent in earlier branching eukaryotes. Thus, several successive evolutionary innovations occurred among their unicellular closest relatives prior to the origin of the multicellular body-plan of animals.

## Introduction

**Figure 1:**
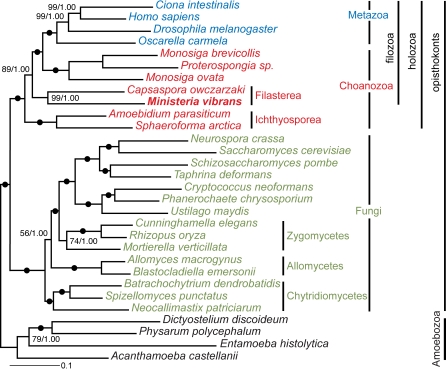
Phylogeny of the Choanozoa and other unikont eukaryotes reconstructed by the maximum likelihood method for 78 protein-coding genes. Numbers beside the internal nodes are maximum likelihood bootstrap values obtained from RaxML and Bayesian MCMC posterior probabilities. Black circles indicate 100% bootstrap support and 1.00 posterior probability values.

Interpretation of molecular phylogeny, morphological and biochemical features suggest that the vast majority of the eukaryote diversity may belong to only six supergroups for review see ref [1]. One of these supergroups, the opisthokonts, is composed of animals, fungi and several smaller groups of unicellular eukaryotes belonging to the phylum Choanozoa [2]–[9]. Recent molecular phylogenetic evidence indicates that animals and fungi evolved independently from different unicellular protozoan choanozoan ancestors [2]–[9]. Choanozoa include both naked phagotrophic protozoa, characterized by long threadlike (filose) cellular projections [3], [10]–[12], often involved in feeding, and others with rigid cell walls, which are saprotrophs or parasites. Of these, nucleariid filose amoebae [13] are probably the closest relatives of fungi [6], [7]. However, the closest relatives of animals were not previously firmly established. Traditionally, one choanozoan lineage, the choanoflagellates, was regarded as most closely related to animals [3]–[4], [14] because some of their tentacles (with a rigid internal skeleton of bundled actin as in animal intestinal microvilli) [15] are aggregated as a collar surrounding the cilium ( = flagellum) in the choanocytes of sponges [16]. A recent multigene analysis showed that choanoflagellates are more closely related to animals than are ichthyosporean Choanozoa [17]. Single gene phylogenies of a broader diversity of Choanozoa raised the possibility that the recently discovered minute marine protozoan *Ministeria vibrans* [10] might be closer still to animals [4], [8]. However those phylogenies were not robust, so it was unclear whether *Ministeria* was sister to animals, or was instead a highly modified choanoflagellate or else (as we show here for the first time) an even deeper branching choanozoan that might give clues as to the nature of still earlier events in the evolution of the unicellular ancestors of animals. To clarify this important evolutionary question we constructed a cDNA library for *M. vibrans* from cultures with both aggregated and dispersed cells to favour the highest possible gene diversity and sequenced 4,700 randomly chosen clones. We have used 78 of the most conserved genes to calculate a more extensive and robust multigene phylogenetic tree than hitherto for opisthokonts (Fig. 1; Supporting Table S1). In addition, we have searched our cDNA sequences for evidence of domains and genes for key animal properties that may have originated in the unicellular ancestors of animals rather than during the origin of multicellularity itself, notably for signaling pathways and cell-adhesion both essential in multicellular animal development.

## Results and Discussion

### Phylogeny of Choanozoa places *Ministeria* as sister to *Capsaspora*

Maximum likelihood (ML) and Bayesian inferences (BI) of a multigene alignment composed of 30 taxa and 17,482 amino acid characters were congruent in showing Amoebozoa (the closest outgroup to opisthokonts) [18], fungi and animals as three distinct clades with maximal statistical support (Fig. 1). Choanozoa in all trees are divided into three distinct lineages with highly supported branching order: 1) choanoflagellates are sisters to animals, 2) the second clade, here designated Filasterea, comprises *Ministeria* and *Capsaspora*, and is robustly sister to choanoflagellates plus animals; 3) Ichthyosporea, comprising *Sphaeroforma* and *Amoebidium*, which are all walled parasites (unlike the phagotrophic choanoflagellates and Filasterea), are robustly sisters to Filasterea plus choanoflagellates and animals (for taxon and genes used in multigene data, see Table S1). Compared with recent evolutionary studies [17], [19], our results greatly improve the resolution and support for the choanozoan branches and show the novel clade Filasterea for the first time. The whole tree is well resolved, with every node having maximal Bayesian support, even among the previously hard to resolve lower fungi where the recent separation of Allomycetes [11] from other Chytridiomycetes is clearly evident. A fusion between ubiquitin and ribosomal small subunit S30 protein once thought animal-specific [20] is here identified in all included choanozoan lineages, thus unifying the holozoa (filozoa plus Ichthyosporea) [5]. Our present analysis leaves undetermined the position of one key free-living choanozoan lineage (*Corallochytrium*, whose cell wall hints at a relationship to Ichthyosoporea, though some trees put it nearer choanoflagellates). No molecular data are available at all for two other putatively choanozoan groups: Fonticulida [3], social amoebae with flat cristae and filopodia that we accordingly place in Discicristoidia, and Aphelidida [21], algal parasites with flat cristae that we place in the parasitic Ichthyosporea. However, sequences might reveal either as a distinct lineage of key significance for eukaryote megaphylogeny.

### Precursors of animal cell adhesion and signaling pathway components among Choanozoa

Polypeptides involved in cell signaling pathways and cell adhesion are essential components in embryogenesis and development of animal body plans [22]–[23]. The majority of the signaling pathways are present across the animal kingdom, even among the primitive sponges that have differentiated cells, epithelia and connective tissue but lack a nervous system or distinct organs [24]–[25]. Domains related to genes in the animal hedgehog, tyrosine kinase and Notch signaling pathways and many adhesion components have also been identified in choanoflagellates, suggesting that some protein domains involved in signaling and cell adhesion originated in the unicellular ancestors of animals [19], [22]. Such signaling and cell adhesion genes are not always composed of domains unique for animals, but may include different combinations of animal novelties and ancestral domains [25]; domains located on the same gene among Eumetazoa can be divided on separate genes in Choanozoa and sponges [19], [26]. It has been suggested that domain shuffling of signaling genes was important in the transition from unicellular eukaryotes to differentiated multicellular animals [19], [26]. Thus, in order to identify precursors of genes and domains involved morphogenesis of animals, it is necessary to apply several approaches that can detect homologies even if sequences lack animal-specific domains [24]–[25], [27]. By using a combination of sequence similarity searches (i.e. BLASTx against NCBInr, NCBIest and Gene ontology databases) and structure analyses against Pfam, InterProScan and Conserved Domain Database, we have annotated several sequences from *Ministeria* and other Choanozoa as signaling and cell adhesion components (Table 1). As in previous studies [24], [27] many of our gene sequences are incomplete, so in some cases we can only be sure that domains homologous with those in related animal genes are present in *Ministeria* (for details, see Supporting Table S2). Nonetheless, there is a remarkably high diversity of domains involved in animal early embryonic development – undoubtedly many more precursors of key animal genes could be found in *Ministeria* if a full genome were sequenced. Annotation of *M. vibrans* cDNA sequences reveals sequences homologous to domains in Notch receptor and ligand (e.g. Notch 1 and 3), hedgehog (Hint domain), and animal tyrosine kinase receptor (e.g. Ros1 protooncogene). Thus, the *Ministeria* data contain domains from the precursors of the hedgehog, tyrosine kinase and Notch pathways, congruent with sequences identified from choanoflagellates [19]. In addition, many components involved in cell adhesion such as crumbs, cadherin, focal adhesion kinase and integrin beta have been identified (Table 1 and Table S2). All these have been identified as animal-specific in recent analyses [19], [24]–[25], [27]. Among other available choanozoan cDNA library sequences we identified similar components from choanoflagellates and *Capsaspora* see also refs. [17] and [19]. Only a few homologues of the signaling and adhesion gene domains could be identified in Ichthyosporea. If a similar paucity is maintained when full genomes are available, this might suggest that most such genes took their modern recognizable form in the last common ancestor of filozoa, after it separated from Ichthyosporea. However, the possibility exists that as Ichthyosporea are parasites they lost some domains secondarily; similar data on the deepest branching Choanozoa, the nucleariid amoeba, are needed to test this. Clearly, some essential prerequisites for development of multicellular organisms and cell differentiation originated earlier than previously thought, distinctly before the origin of the animal kingdom and choanoflagellates.

**Table 1:** Phylogenetic distribution of annotated animal-like signaling and cell adhesion components within Choanozoa.

		Ichthyosporea		Filasterea		Choanoflagellata	
	GO annotation	*Amoebium paraciticum*	*Sphaeroforma arctica*	*Capsaspora owczarzaki*	*Ministeria vibrans*	*Monosiga brevicollis*	*Monosiga ovata*
**Hedgehog signaling**	Hedgehog				•	•	•
	Hyperplastic discs homolog				•		
**Receptor tyrosine kinase signaling**	Ros/insulin family				•		
	Fibroblast growth factor					•	•
	Ephrin type-A receptor						•
	Ephrin type-B receptor						•
**Non-receptor tyrosine kinase signaling**	Abl tyrosine kinase					•	
	Shark			•			
**Notch signaling**	Notch		•		•	•	
	Delta				•		
	ADAM 10				•		
**Cell contact and adhesion proteins**	Integrin-beta				•		
	Crumbs				•		
	Caveolin-1						•
	Focal adhesion kinase 1			•	•	•	•
	Cadherin				•		
	ADAMTS			•			
	Selectin			•			
	Tetraspanin			•			
	Sushi						•
	SHC					•	•
**ECM molecules and receptors**	40S ribosomal protein SA				•		

**Figure 2:**
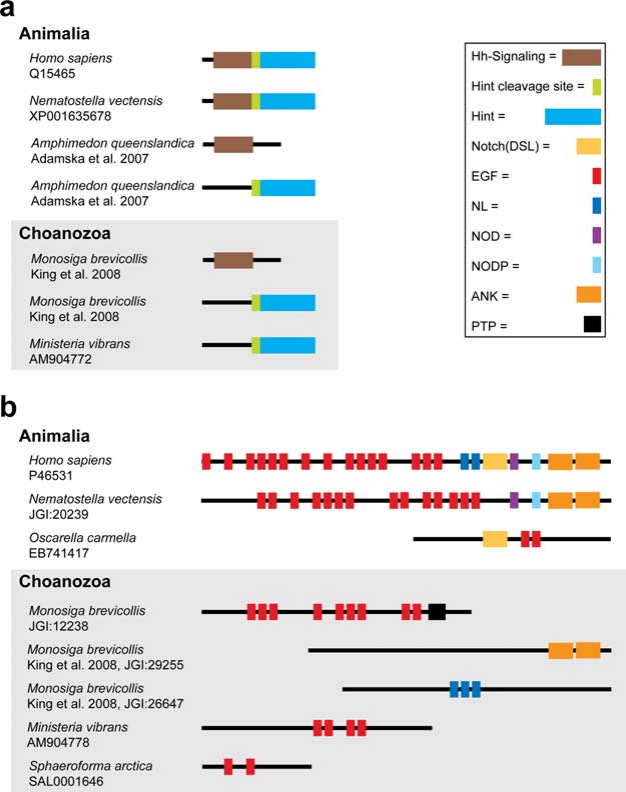
Domain structure of selected annotated sequences. A: hedgehog and B: Notch homologues. The illustrated domains are some of those found by searches against the Conserved Domain Database. Numbers at the species names are accession numbers, protein IDs from the Joint Genome Institute (JGI) and references where annotation recently have been presented. Domain structure identified in *Ministeria* is compared with animals - Porifera (*Amphimedon* and *Oscarella*), Cnidaria (*Nematostella*) and Chordata (*Homo*) - and the choanoflagellate *Monosiga*. Abbreviations: Hh-signal domain, N-terminal hedgehog domain; Hint cleavage site, cleavage site of the C-terminal hedgehog domain; Hint domain, C-terminal hedgehog domain; Notch(DSL), Notch domain also called Delta Serrate Ligand; EGF, epidermal growth factor domain; NL, domain found in Notch and Lin-12; NOD, NOD region; NODP, NODP region; ANK, ankyrin reapeats; PTP, protein tyrosine phosphatase.

As Filasterea are all unicellular, these components must originally have functioned in single cells and were only later co-opted for new functional pathways in multicellular animals. The identified hedgehog relatives in choanoflagellates [19], [28], *Ministeria* and sponges [25] have different domain structures and may illustrate such modification [26], [28]. In Eumetazoa, the hedgehog protein has two main domains, the amino-terminal, signaling domain of hedgehog and the Hint domain, possessing autocatalytic cleavage activity, releasing the signaling domain. Recently, it was found that the hedgehog domains exist in separate genes in the sponge *Amphimedon queenslandica* and the choanoflagellate *M. brevicollis* [19], [26] (Fig. 2). The *Ministeria* polypeptide has a structure only containing the Hint domain and the conserved cleavage motive. Thus, the typical hedgehog domain combination of higher animal genes seems to be absent in Choanozoa and sponges. This also implies that hedgehog domains predate animals and choanoflagellates, and that several successive innovations occurred in this gene family before and after the actual origin of these lineages [9], [19], [26], [28]. The fusion of the two domains in the eumetazoan ancestor changed the transmembrane signaling protein into the diffusible hedgehog ligand of higher animals, enabling signaling over a distance of up to 30 cells [29], a prerequisite for regulation of tissues and organs.

Intriguingly, we could also identify domains partially related to Notch in Ichthyosporea and Filasterea, but the typical N-terminal domains characterized from animal homologues are missing from the sequenced transcripts; even sponges seem to lack many of these domains. If the other Notch domains are present in the genomes of deeply diverging Choanozoa, they may be localized on separate genes similar to what has recently been suggested from genome analysis of choanoflagellates [19]. Our discovery of such a variety of domains that are possible precursors of key animal functions highlights the need for targeted and comparative studies of their functions in Choanozoa and sponges, as recently initiated for a choanoflagellate tyrosine signaling kinase [30].

### Implications on the morphological evolution of early diverging opisthokonts

**Figure 3:**
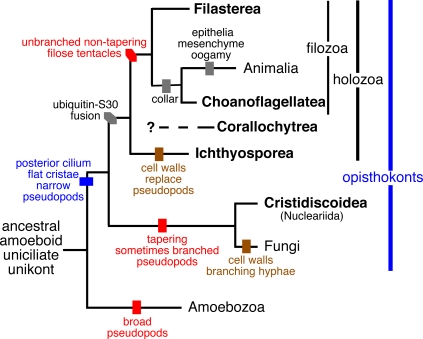
Evolutionary relationships among animals and fungi and their closest unicellular relatives (Choanozoa, Protozoa). The five choanozoan classes (bold) form at least four distinct clades, one probably related to fungi and the others to animals. Innovations in pseudopod character and their multiple losses with the origin of cell walls during nutritional shifts from engulfing prey (phagotrophy) to saprotrophy or parasitism are indicated by bars. In the common ancestor of animals and choanoflagellates a subset of the filozoan actin-supportd tentacles aggregated as a collar around the cilium ( = flagellum) for filter feeding. Epithelia and connective tissue made the first animals: the filter-feeding sponges.

Fig. 3 summaries how several key innovations can be mapped onto the robust phylogeny for the major groups of Choanozoa provided here. Other molecular innovations will only become apparent with full genome sequences of each choanozoan lineage, but can eventually be mapped onto this phylogeny. As Fig. 3 indicates, the origin of slender filose projections, which apparently occurred during the origin of opisthokonts when their ancestor diverged from Amoebozoa (which typically have broad pseudopodia) was probably a key enabling innovation for opisthokont evolution. There is a distinct difference between the non-tapering tentacles of filozoa with their rigid core of bundled actin and the broader and more flexible tapering branched ‘filopodia’ of nucleariids [13]. During the origin of fungi, which probably originally had branched rhizoids as in chytrids [31], the cell wall was probably laid down around such filopodia, which sometimes branch in nucleariids, unlike in Filasterea, thereby creating the ancestor of fungal rhizoids and hyphae. By contrast, in Ichthyosporea and *Corallochytrium* the ancestral filose projections of opisthokonts must have been lost when their cell walls evolved. However, filose tentacles were retained in filozoa for phagotrophic feeding, with a key role, as a filter-feeding collar of tentacles throughout the origin of sponges from choanoflagellate-like ancestors [3].

The grouping of *Ministeria* with *Capsaspora* was unexpected from previous sequence data [4], [8]. As it is fully congruent with their morphological similarities, since *Capsaspora* also has long untapered filose tentacles with a microfilamentous skeleton [32], unlike Ichthyosporea, we establish a new class and separate families for them:

**Taxonomy:** class Filasterea Cavalier-Smith. Diagnosis: naked, unicellular, uninucleate aerobic protozoa, with Golgi dictyosome, flat mitochondrial cristae and very slender long non-tapering, projecting tentacles with an internal skeleton of microfilaments, but which are not organized into a periciliary collar as in choanoflagellates. Trophic phase without wall. Sole order Ministeriida Cavalier-Smith 1997. Family Ministeriidae Cavalier-Smith. Diagnosis: free-living marine filastereans with symmetric radiating tentacles and sometimes vestigial cilium; cysts unknown. Family Capsasporidae Cavalier-Smith. Diagnosis: animal symbionts with lateral tentacles and specialized feeding peduncle; walled resting cyst.

## Materials and Methods

### cDNA library construction and contig assembly

*M. vibrans* (ATCC 50519) cells were grown in ATCC medium 1525 at 17°C. Cells were scraped and harvested by centrifugation and flash frozen in liquid nitrogen. Frozen samples were shipped on dry ice to Agencourt Bioscience Corporation (Beverly MA, USA). The mRNA was isolated and treated with a oligo(dT) -primed procedure for first strand cDNA synthesis. Double strand DNA fragments were directionally ligated into Agencourt pAGEN-1 vector. About 4700 randomly picked clones were sequenced from the 5′-end with the average read length above 600 bp. Sequence contigs were constructed by a Phred/phrap pipeline at the Bioportal service at University of Oslo (http:www.bioportal.uio.no).

### Single- and multigene phylogenetic analyses

Resulting contigs and singletons were screened for sequences usable for phylogeny by BLASTx searches against NCBI nr databases and independent BLASTx searches against manually curated single gene alignments used previously [33]. Sequences from *Ministeria vibrans* and publicly available sequences from the nr- and dbEST databases (http://www.ncbi.nlm.nih.gov/blast/blast_databases.shtml) were retrieved from BLAST results and aligned to a previously published alignment [33] using the Mafft program [34]. For each single gene alignment orthologous gene copies were selected on the basis of phylogenetic relationship in maximum likelihood trees and bootstrap values (i.e. with 100 pseudoreplicates and one tree search for each) inferred with PhyML [35]. Taxa with several almost identical sequences, only the sequence displaying the shortest branch length on the tree was used in subsequent analyses. Among all constructed single gene alignments, we selected 78 genes that had the highest possible coverage of *Ministeria* sequences and that contained at least one sequences from one of choanozoan (i.e. *Amoebidium parasiticum*, *Sphaeroforma arctica*, *Capsaspora owczarzaki*, *Ministeria vibrans*, *Proterospongia sp.* and *Monosiga brevicollis*) lineages. Taxon-sampling were chosen to reflect the phylogenetic range within the opisthokonts (Amoebozoa used as outgroup). All ambiguously aligned sites were deleted before single- and multigene phylogenetic reconstructions. The final concatenated multi-gene matrix included 30 taxa and 17.482 amino acid characters. Each of the included taxa has maximum 70% missing characters (for further information about the taxa and included genes, see supporting Table 1).

Maximum likelihood phylogeny of the concatenated data was inferred with RAxML MPI version 2.2.3 [36]. The rtREV+F evolutionary model was preferred by the ProtTest program [37]. Among site-rate variation were accounted for by using the PROTMIX approximation (initial CAT rate categories set to 25 and final optimization with 4 gamma shape categories) and PROTGAMMA (gamma shaped distribution of site-rates with 4 rate categories) in two separate tree searches. Tree searches were done with 100 randomly generated starting trees. The branching order was identical in both trees inferred with PROTMIX and PROTGAMMA. Furthermore, because of the highly congruent branch pattern from these two runs, the bootstrap analysis was done with the most efficient PROTCAT method (as recommended recently [36]) and rtREV+F on 100 pseudoreplicates and one random starting tree for each replicate.

Bayesian inference was done with PhyloBayes version 2.3 [38]. The CAT evolutionary model was used in together with a gamma distributed across-site variation (4 discrete rate categories). The evolution of the log-likelihood as a function of time was used to estimate if the 2 parallel chains had reached the stationary-state. This was then used to set the burn-in and compare the frequency of the bipartitions between several independent runs. The largest discrepancy (maxdiff) between the bipartitions was less than 0.1 and therefore we considered the Markov chain Monte Carlo chains to have converged. The tree and posterior probability values presented in Fig. 1 are a consensus of the cold chains from the 2 independent runs. All phylogenetic analyses were performed on the freely available Bioportal at Universtity of Oslo (http://www.bioportal.uio.no).

### Identification of precursors of animal signaling and cell adhesion domains among Choanozoa

All contigs and singletons generated from the *M. vibrans* cDNA library and other available Choanozoa sequences were annotated in order to identify homologs of previously defined signaling and cell adhesion protein components that are involved in morphogenesis of animals and only identified in the animal kingdom and the choanoflagellates [19], [25]–[26], [28]. Thus, although components involved in for instance tyrosine kinase signaling pathways have been identified outside the Animalia, we only searched for types of components that have been suggested as animal-specific [19], [25]–[26], [28]. First, the sequences were screened against the NCBInr protein databases using BLASTx. All query sequences that retrieved relevant genes were further annotated by searches against the Gene Ontology (GO) database (http://amigo.geneontology.org/cgi-bin/amigo/go.cgi). A similar procedure was recently applied for sponge cDNA sequences [25]. For each search only the GO descriptions with highest e-values were considered and assembled in a local database (Table 1 and Table S2). These sequences were subsequently searched against several motif and domain databases (Fig. 2) including InterProScan (http://www.ebi.ac.uk/InterProScan/) and Conserved Domain database (http://www.ncbi.nlm.nih.gov/sites/entrezdbcdd). Furthermore, in order to uncover protein domains in other genes than those identified in Table 1, single domains from human Hedgehog, Notch and Delta genes were used as query in BLAST searches (i.e. BLASTp and tBLASTn) against the NCBI databases (i.e. NCBInr and NCBIest) and local databases comprising the *Ministeria vibrans* cDNA and sequences downloaded from the Protist EST Project (PEP; http://www.bch.umontreal.ca/pepdb/pep_main.html).

## Supporting Information

Table S1 (0.96 MB PDF)

Table S2 (0.08 MB PDF)
