# Supplementary material for: Multigene Phylogeny of Choanozoa and the Origin of Animals
Source: PLoS One. 2008 May 7;3(5):e2098. doi: 10.1371/journal.pone.0002098 (PMC2346548; doi:10.1371/journal.pone.0002098)
Supplement: Table S1 — (0.96 MB PDF) [file pone.0002098.s001.pdf]

Supporting Information

Multigene phylogeny of Choanozoa and the origin of animals

Kamran Shalchian-Tabrizi, Marianne A. Minge, Mari Espelund, Russell Orr,

Torgeir Ruden, Kjetill S. Jakobsen, Thomas Cavalier-Smith

Supporting Table S1. Taxa and genes used in the phylogenetic analyses

	Acanthamoeba castellanii	*Allomyces macrogynas*	*Amoebidium parasiticum*	*Batrachochytrium dendrobatidis*	*Blastocladiella emersonii*	*Capsaspora owzarzaki*	*Ciona intestinalis*	*Cryptococcus neoformans*	*Cunninghamella elegans*	*Dictyostelium discoideum*	*Drosophila melanogaster*	*Entamoeba histolytica*	*Homo sapiens*	*Ministeria vibrans*	*Monosiga brevicollis*	*Monosiga ovata*	*Mortierella verticillata*	*Neocallimastix patriciarum*	*Neurospora crassa*	*Oscarella carmela*	*Phanerochaete chrysosporium*	*Physarum polysephalum*	*Proterospongia sp.*	*Rhizopus oryzae*	*Saccharomyces cerevisiae*	*Schizosaccharomyces pombe*	*Sphaeroforma arctica*	*Spizellomyces punctatus*	*Taphrina deformans*	*Ustilago maydis*
AAA-ATPase nsf1-G	x				x	x	x	x		x	x	x	x	x	x	x	x		x		x				x	x		x		x
AAA-ATPase nsf1-I		x		x	x	x	x	x		x			x	x		x	x		x		x	x		x	x	x		x		x
AAA-ATPase nsf1-J				x	x	x	x	x		x			x	x	x	x		x	x		x		x	x	x	x	x	x		x
AAA-ATPase nsf1-K			x	x	x	x	x	x		x			x	x	x				x		x			x	x	x				x
AAA-ATPase nsf1-M		x		x	x		x	x		x	x	x	x			x	x		x	x	x	x		x	x	x				x
AAA-ATPase nsf2-A	x			x			x	x		x	x		x	x		x	x		x		x		x	x	x	x		x		x
AAA-ATPase nsf2-F				x			x	x		x	x		x	x					x	x	x	x			x	x				x
Actin	x	x	x	x	x	x	x	x	x	x		x	x	x	x	x	x	x	x	x	x	x	x	x	x	x	x	x	x	x
Cytosolic chaperonin A				x		x	x		x	x			x	x					x		x	x	x	x	x	x				x
Cytosolic chaperonin B	x			x	x	x	x	x	x	x	x	x	x		x	x	x		x	x	x	x		x	x	x	x			x
Cytosolic chaperonin D				x	x		x	x		x	x	x	x	x			x		x	x	x	x		x	x	x	x	x		x
Cytosolic chaperonin E	x		x	x	x	x	x	x	x	x	x	x	x	x	x	x	x		x	x	x	x			x	x	x			x
Cytosolic chaperonin N		x		x	x	x	x	x	x	x	x			x	x	x	x		x	x	x	x			x	x	x	x		x
Cytosolic chaperonin T				x	x		x	x	x	x	x	x	x	x		x			x		x	x			x	x		x		x
Cytosolic chaperonin Z	x			x	x	x	x	x	x	x	x		x	x				x	x	x	x				x	x	x	x		x
Elongation factor 1	x		x	x			x	x	x	x	x	x	x	x		x	x	x	x	x	x	x		x	x	x			x	x
Elongation factor 2		x	x	x	x		x	x	x	x	x	x	x	x	x	x	x	x	x		x	x		x	x	x		x		x
Eukaryotic initiation factor 4a		x		x	x			x	x		x		x	x		x	x	x	x	x		x	x	x		x	x	x		x
Fibrillarin	x				x		x	x	x	x	x	x	x	x	x		x		x	x	x	x	x		x	x	x	x	x	x
Heat shock protein 90	x	x		x	x				x	x	x	x	x	x		x	x	x	x	x		x		x	x	x	x	x		
Initiation factor 1 g		x		x	x	x	x		x	x	x	x	x	x	x	x			x		x	x		x	x	x	x	x	x	x
Initiation factor 1 p	x			x			x	x	x		x	x	x	x				x	x		x	x			x	x		x		x
Initiation factor 6	x	x		x	x	x	x	x	x	x	x	x	x			x	x	x	x		x			x	x	x				x
Large-subunit ribosomal protein 1	x	x	x	x	x	x	x	x	x	x	x	x	x		x	x	x	x	x	x	x	x	x	x	x	x	x	x	x	x
Large-subunit ribosomal protein 2	x	x	x	x	x	x	x	x	x	x	x	x	x	x	x	x	x	x	x	x	x	x		x	x	x	x	x	x	x
Large-subunit ribosomal protein 3	x	x	x	x	x	x	x	x	x	x	x		x	x	x	x	x	x	x	x	x	x		x	x	x	x	x	x	x
Large-subunit ribosomal protein 4b	x	x	x	x	x	x	x	x	x	x	x		x	x	x	x	x	x	x		x	x	x	x	x	x	x	x	x	x
Large-subunit ribosomal protein 5	x	x	x	x	x	x	x	x	x	x	x	x	x	x	x	x	x	x	x	x	x			x	x	x	x	x	x	x
Large-subunit ribosomal protein 6				x		x	x	x		x	x		x	x	x	x	x	x	x		x			x	x	x	x	x		x
Large-subunit ribosomal protein 7a	x	x		x		x	x	x	x	x	x	x	x		x	x	x	x	x	x	x		x	x	x	x	x	x	x	x
Large-subunit ribosomal protein 9	x	x	x	x	x	x	x	x	x	x	x	x	x		x	x	x	x	x		x	x	x	x	x	x	x	x	x	x
Large-subunit ribosomal protein 11b	x	x	x	x	x	x	x	x	x	x	x	x	x		x	x	x	x	x	x	x	x	x	x	x	x	x	x	x	x
Large-subunit ribosomal protein 12b	x	x	x	x	x	x	x	x	x	x	x	x	x		x	x	x	x	x	x	x			x	x	x	x		x	x
Large-subunit ribosomal protein 13	x	x		x	x	x	x	x	x	x	x	x	x		x	x	x	x	x		x		x	x	x	x	x	x	x	x
Large-subunit ribosomal protein 14	x		x			x	x	x		x	x		x	x		x	x		x		x			x	x	x	x	x		x
Large-subunit ribosomal protein 15	x	x	x	x	x	x	x	x	x	x	x	x	x				x	x	x		x	x		x	x	x	x	x	x	x
Large-subunit ribosomal protein 16	x	x	x	x	x	x	x	x	x	x	x		x		x	x	x	x	x	x	x	x	x	x	x	x	x	x	x	x
Large-subunit ribosomal protein 17	x	x		x		x	x	x	x	x	x	x	x	x	x	x		x	x	x	x	x	x	x	x	x	x	x	x	x
Large-subunit ribosomal protein 18	x	x		x	x	x	x	x	x	x	x		x		x	x	x	x	x	x	x		x	x	x	x	x	x	x	x
Large-subunit ribosomal protein 19	x	x		x	x	x	x	x	x	x	x	x	x		x	x	x	x	x			x	x	x	x	x	x	x	x	x
Large-subunit ribosomal protein 20	x	x	x	x	x	x	x	x	x	x	x		x		x	x	x		x	x	x			x	x	x	x	x		x
Large-subunit ribosomal protein 27	x	x	x	x	x	x	x	x		x	x	x	x		x	x	x	x	x		x	x	x	x	x	x	x	x	x	x
Large-subunit ribosomal protein 30	x	x	x	x	x	x	x	x	x	x	x	x	x	x	x	x	x		x		x			x	x	x	x	x	x	x
Large-subunit ribosomal protein 32	x	x	x	x	x	x	x	x	x	x	x	x	x	x	x	x	x		x		x	x		x	x	x	x	x	x	x
Large-subunit ribosomal protein 35	x			x		x	x	x	x	x	x	x	x			x	x	x	x	x	x	x		x	x	x	x	x	x	x
Large-subunit ribosomal protein 37	x	x	x	x	x	x	x	x	x	x	x	x	x			x	x	x	x		x			x	x	x	x	x	x	x
Large-subunit ribosomal protein 42	x	x	x	x	x	x	x	x		x	x		x		x	x	x	x	x		x	x	x	x	x	x	x	x	x	x
Large-subunit ribosomal protein 43	x	x	x	x	x	x	x		x	x	x		x		x	x		x	x		x	x		x	x	x	x	x	x	x
Methionine adenosyltransferase 1				x	x	x	x	x	x	x	x	x	x	x	x	x		x	x	x		x		x	x	x		x	x	x
Mitochondrial chaperonin 60					x		x	x	x	x	x	x	x	x	x	x			x	x		x		x	x	x	x	x	x	x
Proteasome AAA-ATPase reg. su D	x	x		x	x	x	x	x		x	x	x	x			x	x		x		x			x	x	x				x
Proteasome AAA-ATPase reg. su E	x	x		x	x	x	x	x		x	x	x	x			x	x		x		x			x	x	x	x	x		x
Ribosomal protein L10 (grc5)	x	x	x	x	x	x	x	x	x	x	x	x	x		x	x	x	x	x	x	x	x	x	x	x	x	x	x	x	x
Ribosomal protein p0	x	x		x	x	x	x	x	x	x	x	x	x	x	x	x	x	x	x	x	x	x	x	x	x	x	x	x	x	x
Ribosomal protein S12/L30/L7a family A		x		x	x		x	x	x	x	x		x	x			x	x	x		x	x		x	x	x		x		x
Ribosomal protein S2	x	x	x	x	x	x	x	x	x	x	x	x	x	x	x	x	x	x	x	x		x	x	x	x	x	x	x	x	x
Seryl tRNA synthetase	x			x			x			x	x		x	x							x			x	x	x				
Small-subunit ribosomal protein 1	x	x	x	x	x	x	x	x	x	x	x		x		x	x	x		x	x	x	x	x	x	x	x	x	x	x	x
Small-subunit ribosomal protein 2	x	x	x	x	x	x	x	x	x	x	x	x	x	x	x	x	x	x	x	x	x	x		x		x	x	x	x	x
Small-subunit ribosomal protein 3	x	x		x		x	x	x			x	x	x	x	x	x	x		x		x	x	x	x	x	x	x	x	x	x
Small-subunit ribosomal protein 4	x	x	x	x	x	x	x	x	x	x	x	x	x		x	x	x	x	x	x	x	x		x	x	x	x	x	x	x
Small-subunit ribosomal protein 5	x	x	x	x	x	x	x	x	x	x	x	x	x	x	x	x	x	x	x		x	x	x	x	x	x	x	x	x	x
Small-subunit ribosomal protein 6	x	x	x	x	x	x	x	x	x	x	x	x	x	x	x	x	x	x	x		x	x	x	x	x	x	x	x	x	x
Small-subunit ribosomal protein 8	x	x	x	x	x		x	x	x	x	x		x		x		x	x	x	x	x		x	x	x	x	x	x	x	x
Small-subunit ribosomal protein 10				x		x	x	x		x	x	x	x	x	x		x	x	x		x	x		x	x	x	x	x		x
Small-subunit ribosomal protein 11	x	x			x	x	x	x	x	x			x	x	x	x	x	x	x	x	x	x			x	x	x	x		x
Small-subunit ribosomal protein 14		x	x	x	x	x	x	x		x	x	x	x	x	x	x	x		x	x	x	x	x	x	x	x	x	x	x	x
Small-subunit ribosomal protein 16	x	x	x	x	x	x	x	x	x	x	x	x	x		x	x	x	x	x	x	x	x	x	x	x	x	x	x	x	x
Small-subunit ribosomal protein 18	x	x		x	x	x	x	x	x	x	x	x	x			x	x	x	x		x	x	x	x	x	x	x	x	x	x
Small-subunit ribosomal protein 22a	x	x	x	x	x		x	x	x	x	x	x	x		x		x	x	x		x	x	x	x	x	x	x	x	x	x
Small-subunit ribosomal protein 23	x	x	x		x	x	x	x	x	x			x		x	x	x	x	x		x	x	x	x	x	x	x	x	x	
Succinate CoA ligase		x			x	x	x	x	x	x	x	x	x		x	x		x	x			x		x	x	x	x	x		x
Threonyl-tRNA synthetase	x	x		x			x			x			x	x								x			x	x		x		x
Topoisomerase I							x	x	x	x			x	x		x			x		x				x	x				
Tubulin beta	x	x		x	x		x	x		x	x	x	x	x	x	x	x		x	x	x	x	x		x	x	x	x	x	x
V-ATPase subunit A					x		x	x	x	x	x	x	x	x	x	x			x		x	x		x	x	x				
V-ATPase subunit B				x	x		x	x	x	x	x	x	x	x	x	x			x	x	x	x		x	x	x				x
V-ATPase subunit E				x	x	x	x	x	x	x	x		x		x				x		x			x	x	x				x
% missing data	48.2	51.5	67.8	17.1	32.6	48.6	5.2	5.4	31.8	4.8	2.9	39.9	0.0	43.4	40.4	26.1	39.7	39.7	1.6	60.1	18.3	32.4	69.6	28.4	3.4	0.0	47.3	43.1	59.2	5.0
