# Supplementary material for: Multigene Phylogeny of Choanozoa and the Origin of Animals
Source: PLoS One. 2008 May 7;3(5):e2098. doi: 10.1371/journal.pone.0002098 (PMC2346548; doi:10.1371/journal.pone.0002098)
Supplement: Table S2 — (0.08 MB PDF) [file pone.0002098.s002.pdf]

Supporting Information

Multigene phylogeny of Choanozoa and the origin of animals

Kamran Shalchian-Tabrizi, Marianne A. Minge, Mari Espelund, Russell Orr,

Torgeir Ruden, Kjetill S. Jakobsen, Thomas Cavalier-Smith

Supporting Table S2. Phylogenetic distribution of sequences with homology to animal signaling and cell adhesion components.

Component function	Organism	EST-ID: GO annotation and E value
**Hedgehog signaling**		
Hedgehog (Hint domain)	*M. vibrans*	AM904772: Qua-1 7.5e-9
Hoglet	*M. ovata*	MNL00000104: Shha 1.2e-08
Hyperplastic discs homolog	*M. vibrans*	AM904773: Hyperplastic discs homolog 1e-8
**Receptor tyrosine kinase signaling**		
Ros/insulin family	*M. vibrans*	AM904774: Ros1 protooncogene 2.2e-15
Fibroblast growth factor	*M. brevicollis*	MRL00000285: Basic fibroblast growth factor receptor 1 precursor 2.6e-40
Fibroblast growth factor	*M. ovata*	MNL00000545: Acidic fibroblast growth factor intracell.-binding protein 1.4e-30
Ephrin type-A receptor	*M. ovata*	MNL00001478: eph receptor A4a 1.1e-17
Ephrin type-B receptor	*M. ovata*	MNL00002676: Ephrin type-B receptor 1 precursor 4.6e-25
Ephrin type-B receptor	*M. ovata*	MNL00002507: Eph receptor B3 1.6e-08
**Non-receptor tyrosine kinase signaling**		
Abl tyrosine kinase	*M. brevicollis*	MRL00000743: Abl tyrosine kinase 4.6e-18
Shark	*C. owczarzaki*	NUL00000096: shark, Src homology 2, ankyrin repeat, tyrosine kinase 1.1e-15
**Notch signaling**		
Notch 1	*M. vibrans*	AM949835: Notch 1 2.6e-80
Notch 1b	*M. vibrans*	AM949836: Notch 1b 1.1e-81
Notch 1b	*M. vibrans*	AM950290: Notch homolog 1b precursor 6.6e-75
Notch 3	*S. arctica*	SAL00001646: Neurogenic locus Notch homolog protein 3 precursor 3.0e-14
Notch	*M. vibrans*	AM904778: Notch homolog 3 2.0e-56
Notch	*M. brevicollis*	JGI 12238: 1.4e-131
Delta	*M. vibrans*	AM904779: dlc, deltaC 4.9e-42
ADAM 10	*M. vibrans*	AM904780: ADAM 10 4.6e-35
**Cell contact and adhesion proteins**		
Integrin-beta	*M. vibrans*	AM904781: Integrin-beta-like 2.5e-19
Crumbs	*M. vibrans*	AM949838: Crumbs homolog 2 3.6e-75
Caveolin-1	*M. ovata*	MNL00000290: Caveolin, caveolae protein 1 1.3e-09
Focal adhesion kinase 1	*C. owczarzaki*	NUL00001927: Focal adhesion kinase 1 5.3e-22
Focal adhesion kinase 1	*M. vibrans*	AM904783: Focal Adhesion Kinase 1.6e-51
Focal adhesion kinase 1	*M. brevicollis*	MRL00000384: Focal adhesion kinase 1 1.0e-07
Focal adhesion kinase 1	*M. ovata*	MNL00002118: Focal adhesion kinase 1 2.0e-19
Cadherin	*M. vibrans*	AM904784: Cadherin-24 1.9e-7
ADAMTS	*C. owczarzaki*	NUL00000036: ADAMTS-like protein 4 precursor 3.9e-36
Selectin	*C. owczarzaki*	NUL00000949: P-selectin precursor 9.2e-16
Tetraspanin	*C. owczarzaki*	NUL00000822: Tetraspanin-31 5.0e-31
Sushi	*M. ovata*	MNL00000784 Csmd1, CUB and Sushi multiple domains 1 3.8e-07
SHC	*M. brevicollis*	MRL00001066: SHC-transforming protein 2 4.7e-09
SHC	*M. ovata*	MNL00001125: SHC-transforming protein 2 3.1e-52
SHC	*M. ovata*	MNL00001320: SHC-transforming protein 1 3.1e-16
**ECM molecules and receptors**		
40S ribosomal protein SA	*M. vibrans*	AM904785: 40S ribosomal protein SA 3.8e-80
